# Short-form video addiction and psychological resilience: a cognitive-existential serial mediation model among Chinese college students

**DOI:** 10.3389/fpsyg.2026.1846754

**Published:** 2026-06-11

**Authors:** Xiaowei Li, Pan Chen, Wei Tang, Peng Lei

**Affiliations:** 1 School of Marxism, Sichuan Technology and Business University, Chengdu, China; 2 School of Tourism and Cultural Industries, Sichuan Tourism University, Chengdu, China; 3 School of Economics, Southwestern University of Finance and Economics, Chengdu, China

**Keywords:** belief in a just world, Chinese college students, meaning in life, psychological resilience, serial mediation, short-form video addiction

## Abstract

This study examined the serial mediating roles of belief in a just world (BJW) and meaning in life in the association between short-form video addiction and psychological resilience among Chinese college students. A total of 4,456 participants (*M*age = 20.34, *SD* = 1.52; 52.1% female) from five universities in Sichuan Province completed measures of short-form video addiction, BJW, meaning in life, and psychological resilience. Serial mediation analysis (PROCESS Model 6; 5,000 bootstrap samples) revealed that short-form video addiction negatively predicted resilience (*B* = −0.1948, *p* < 0.001). Three indirect pathways were significant: through BJW alone [Effect = −0.0337, 95% CI (−0.0426, −0.0254)], through meaning in life alone [Effect = −0.0606, 95% CI (−0.0769, −0.0443)], and through BJW sequentially to meaning in life [Effect = −0.0274, 95% CI (−0.0337, −0.0215)]. The total indirect effect accounted for 62.5% of the total effect. Meaning in life was the strongest proximal correlate of resilience (*β* = 0.5410). These findings are consistent with a cognitive-existential sequential association pattern in which short-form video addiction is associated with weakened worldview beliefs and diminished existential meaning, which in turn are associated with lower resilience. Implications for multi-layered university interventions are discussed.

## 1 Introduction

### 1.1 Research background and problem statement

Short-form video platforms such as TikTok (known as Douyin in China), Kuaishou, and Bilibili have experienced explosive growth over the past several years, fundamentally reshaping digital media consumption patterns among young adults worldwide. As of June 2024, the number of short-form video users in China surpassed 1.05 billion, representing approximately 95.5% of all internet users across all age groups (China Internet Network Information Center, 2024). This near-universal penetration reflects the extent to which short-form video consumption has become embedded in daily digital life. It is important to note, however, that high prevalence of use does not in itself imply psychological harm: the majority of users engage with these platforms in normative, adaptive ways. The critical concern lies with the subset of users whose engagement escalates into addictive or problematic patterns—a minority in proportional terms, but one whose absolute numbers are substantial given the scale of the overall user base, and whose psychological well-being warrants dedicated empirical attention.

College students constitute a particularly active user base and face heightened vulnerability both to developing addictive use patterns and to experiencing adverse psychological consequences from such patterns, given the developmental characteristics of emerging adulthood. Surveys consistently indicate that over 80% of Chinese university students regularly engage with short-form video content (Liu, 2019), and users aged 20 to 29 account for the largest share of highly educated short-form video consumers (Vailshery, 2025). At the platform level, highly personalized algorithmic recommendation systems and ultra-brief, high-stimulation content formats are specifically engineered to maximize engagement through variable reward schedules that trigger dopaminergic pathways (Tian et al., 2023; Wise and Robble, 2020). These design features, combined with infinite scrolling interfaces that minimize natural stopping points, create conditions uniquely conducive to compulsive use.

Recent systematic reviews have identified multiple psychological antecedents and consequences of short-form video addiction among Chinese university students, including depression, anxiety, loneliness, and impaired academic functioning (Ye et al., 2022; Zhan and Zhu, 2025). A recent study of 11,425 Chinese college students further documented that adverse childhood experiences increase the risk of short-form video addiction, with resilience and life satisfaction serving as mediating buffers (Xue et al., 2025). Despite this growing awareness, the specific cognitive and existential pathways through which short-form video addiction may be associated with adaptive psychological capacities—particularly psychological resilience—remain insufficiently explored.

Psychological resilience, broadly defined as the capacity to adapt successfully and maintain positive functioning in the face of adversity, stress, or significant challenge (Masten, 2001), is widely recognized as a critical protective factor for college students navigating the developmental demands of emerging adulthood. Resilience enables individuals to cope with academic pressures, manage interpersonal conflicts, recover from setbacks, and sustain psychological well-being during periods of transition and uncertainty (Connor and Davidson, 2003; Luthar et al., 2000). Meta-analytic evidence has confirmed that trait resilience is positively associated with positive mental health indicators and negatively associated with psychological distress across diverse populations and measurement approaches (Hu et al., 2015). Within the specific domain of digital media, an emerging literature has documented associations between various forms of technology overuse and resilience-related outcomes: a recent chain mediation study found that resilience and self-control serially mediated the relationship between proactive personality and short-form video addiction among Chinese college students (Wu et al., 2024), and research on adverse childhood experiences has demonstrated that resilience and life satisfaction jointly mediate pathways from early adversity to short-form video addiction (Xue et al., 2025). However, the direction of inquiry in these studies—treating resilience as a predictor or mediator of addiction rather than as an outcome that addiction may be associated with—addresses a different theoretical question from that examined in the present study. Grounded in Conservation of Resources (COR) theory (Hobfoll, 1989), the present study conceptualizes addictive engagement with short-form video as a resource-depleting behavioral pattern, proposing that the resulting erosion of cognitive, temporal, and emotional reserves may be associated with downstream diminishment in adaptive capacity—of which psychological resilience is a central indicator. This directional framing, in which sustained resource-depleting behavior is associated with deterioration in adaptive functioning, is foundational to COR theory and provides a theoretical basis for treating psychological resilience as an outcome variable in the current model. Furthermore, emerging adulthood represents a developmentally sensitive period during which key psychological resources, including resilience, are actively being consolidated, making this population particularly relevant for examining how addictive media engagement may be associated with adaptive capacity.

While prior research has examined various mediating processes in digital addiction contexts—including rumination, social comparison, and self-regulation failure—the specific serial pathway through which short-form video addiction may be associated with resilience via cognitive worldview beliefs and existential meaning has not been tested. This represents a significant theoretical gap: without understanding these mediating associations, it is difficult to identify the most effective intervention targets or to design preventive strategies that address root causes rather than surface symptoms. To address this gap, the present study examines an integrated serial mediation model in which belief in a just world and meaning in life serve as sequential mediators linking short-form video addiction to psychological resilience.

### 1.2 Theoretical framework and hypotheses development

The present study integrates Conservation of Resources (COR) theory as the overarching motivational framework with two domain-specific theories addressing cognitive and experiential processes. COR theory (Hobfoll, 1989) provides the foundational logic: individuals strive to acquire, protect, and accumulate valued psychological resources, and the depletion of these resources under conditions of addictive digital engagement is associated with deterioration in adaptive functioning. Within this framework, belief in a just world (BJW) represents a cognitive resource grounded in Just World Theory (Dalbert, 2001; Lerner, 1980), while meaning in life represents an experiential resource grounded in existentialist theory (Frankl, 1985; Steger et al., 2006). Both resources are proposed to operate sequentially, consistent with cognitive coherence theory’s proposition that worldview-level beliefs provide epistemic foundations for constructing personal meaning (Heintzelman and King, 2014). The specific hypotheses are developed below.

#### 1.2.1 Short-form video addiction and psychological resilience

Within the COR framework, addictive engagement with short-form videos may be associated with the depletion of psychological resources across multiple domains simultaneously. Temporally, compulsive viewing may displace time allocated to academic work, face-to-face social interaction, physical exercise, and restorative sleep. Cognitively, the rapid-fire nature of algorithmically curated content may be associated with fragmented attention and impaired executive functioning, which represent foundational capacities for effective stress coping (Chen et al., 2023; Xie et al., 2023). Emotionally, habitual reliance on short-form videos as a primary affect regulation strategy may attenuate the development of more adaptive coping repertoires. These patterns of resource depletion are particularly consequential for resilience, which depends upon the availability and integration of cognitive, emotional, and social resources (Masten, 2001); as addictive engagement is associated with progressive erosion of these resources, the adaptive capacity underpinning resilience may correspondingly diminish. Short-form video platforms possess design features—highly personalized algorithmic recommendation, ultra-brief and high-stimulation content, infinite scrolling interfaces—that may render them uniquely conducive to compulsive use relative to traditional social media or other digital technologies (Zhan and Zhu, 2025). Based on this reasoning, we hypothesize:

*H1*: Short-form video addiction is negatively associated with psychological resilience.

#### 1.2.2 The mediating role of belief in a just world

Belief in a just world (BJW) refers to the fundamental cognitive assumption that the world operates according to principles of fairness and that people generally receive the outcomes they deserve (Lerner, 1980). BJW functions as a personal resource by providing individuals with a sense of predictability, control, and existential security (Dalbert, 2001). When individuals endorse the view that the world is just, they are more inclined to appraise stressful events as controllable challenges rather than uncontrollable threats, facilitating the adoption of active and problem-focused coping strategies (Dalbert, 1999; Lazarus, 1984). Extensive empirical evidence supports the adaptive functions of BJW: personal BJW is associated with greater well-being, lower depressive affect, and more positive mental health outcomes across diverse populations (Hafer and Sutton, 2016), with perceived control and optimism identified as key mediating mechanisms (Goodwin and Williams, 2023). Critically for the present study, Wu et al. (2011) demonstrated in a Chinese collectivist sample that general BJW was positively associated with resilience, providing direct cross-cultural evidence for the BJW-resilience link. Wang et al. (2023) further confirmed BJW’s adaptive behavioral functions in a Chinese college student sample, demonstrating that higher BJW positively predicted prosocial outcomes, underscoring its role as a positive psychological resource within this specific population.

Several theoretically proposed pathways suggest how short-form video addiction may be associated with weakened BJW. First, one plausible theoretical account involves the algorithmic architecture of short-form video platforms. It has been theoretically proposed that such platforms may disproportionately amplify emotionally provocative and extreme content, including depictions of social injustice, exploitation, and systemic inequality, given that such content tends to generate higher engagement metrics. Repeated and prolonged exposure to such algorithmically curated injustice-related content could, under this account, be associated with progressive erosion of the belief that the world operates fairly. It should be noted, however, that the present study did not include direct measures of content exposure types or users’ subjective experience of algorithmic curation; this proposed account therefore remains a theoretical inference that awaits direct empirical testing. As Goodwin and Williams (2023) noted, general BJW may be particularly sensitive to media exposure because it reflects beliefs about the broader world rather than one’s personal experience—a characteristic that renders it theoretically susceptible to the cumulative influence of content patterns encountered through sustained media engagement. Second, short-form videos frequently showcase conspicuous displays of wealth, attractiveness, and effortless success, potentially triggering upward social comparison processes and generating perceptions of distributive unfairness (Vogel et al., 2014). Third, the experience of addiction itself may produce cognitive dissonance: individuals who recognize that they cannot control their viewing behavior despite wanting to may experience a self-directed perception of unfairness that could generalize to broader worldviews (Festinger, 1957). To the extent that these theoretically proposed pathways operate as described, the cognitive resources available for resilient adaptation—including the sense of predictability, control, and fairness that supports active coping—may be correspondingly reduced. Therefore, we hypothesize:

*H2*: Belief in a just world mediates the relationship between short-form video addiction and psychological resilience (i.e., Video → BJW → PR).

#### 1.2.3 The mediating role of meaning in life

Meaning in life refers to the subjective experience of one’s existence as purposeful, significant, and coherent (Steger et al., 2006). Within the existentialist psychological tradition, meaning has been conceptualized as a fundamental human need whose fulfillment is essential for psychological health and adaptive functioning (Frankl, 1985). Self-determination theory further posits that meaning emerges from the satisfaction of basic psychological needs for autonomy, competence, and relatedness (Ryan and Deci, 2000). Meaning in life has been empirically linked to a broad range of positive outcomes, including greater psychological well-being, lower levels of depression and anxiety, and enhanced resilience (Park, 2010; Steger and Kashdan, 2007). A recent meta-analysis of 228 studies confirmed medium-level positive correlations between meaning in life and positive mental health indicators among college students (Zhan and Zhu, 2025). Moreover, a growing body of evidence specifically documents the relationship between meaning and resilience: a recent comprehensive review concluded that meaning in life is associated with resilience through self-regulatory processe**s** including stress buffering, adaptive coping facilitation, and healthy behavior engagement (Miao and Cao, 2024), and empirical research has demonstrated that presence of meaning (as operationalized by the MLQ Presence subscale) mediates the relationship between mindfulness and resilience in undergraduates (Hooker et al., 2018).

Short-form video addiction may be associated with diminished meaning in life through several theoretically proposed pathways. The most direct pathway involves temporal displacement: compulsive viewing may reduce participation in inherently meaningful activities such as deep learning, creative expression, community engagement, and intimate social interaction, all of which are primary sources of existential meaning (Steger et al., 2008). Additionally, the passive consumption model that characterizes short-form video engagement stands in contrast to the active, autonomous engagement with the world that generates meaning. Although short-form videos provide immediate hedonic gratification, this ephemeral pleasure may lack the depth and coherence necessary to sustain enduring purpose. A recent study documented this pattern, finding that short video addiction was associated with adolescents’ well-being through a chain mediation involving emotional deterioration and loss of life meaning (Liu et al., 2025). Finally, repeated immersion in fragmentary, superficial content may foster broader existential emptiness—the recognition that significant portions of finite time are being consumed by activities one does not regard as genuinely worthwhile. Therefore, we hypothesize:

*H3*: Meaning in life mediates the relationship between short-form video addiction and psychological resilience (i.e., Video → Life → PR).

#### 1.2.4 The serial mediation: from cognition to meaning

Beyond functioning as parallel mediators, BJW and meaning in life may operate in a sequential chain in which cognitive beliefs about the world’s fairness may function as an antecedent factor associated with existential meaning. This proposition aligns with cognitive-experiential models suggesting that foundational worldviews shape the quality of subjective experience (Beck, 1976). BJW represents a meta-cognitive belief about the structure and predictability of the social world. When individuals believe the world is fundamentally just, they may more readily trust that their efforts will be rewarded, their goals are worth pursuing, and their lives unfold according to comprehensible principles. This sense of cosmic orderliness may provide cognitive scaffolding upon which existential meaning is more readily constructed (Heintzelman and King, 2014). Conversely, when BJW is weakened, the perceived randomness and unfairness of the world may be associated with reduced confidence in goal pursuit, diminished sense of effort’s value, and uncertainty about life purpose.

Empirical evidence increasingly supports this directional relationship. Tian et al. (2022) found that both the presence of and search for meaning in life mediated the relationship between BJW and mental toughness—a construct related to but conceptually distinct from psychological resilience, referring in that study to the capacity of adolescent athletes to maintain performance under competitive pressure; we cite this finding specifically as evidence for the proposed directional association between BJW and meaning in life, rather than as direct evidence regarding the resilience outcome examined here—among 1,544 Chinese adolescent athletes, with the presence of meaning playing a more influential role. More recently, Zhou and Toikko (2025) demonstrated in a sample of 2,075 Chinese young adults that presence of meaning in life mediated the relationship between personal BJW and mental health outcomes, including positive emotions and reduced psychological symptoms. These findings suggest that BJW may be associated with psychological adjustment partly through its association with existential meaning construction.

We therefore propose a sequential association pattern consistent with the following process: short-form video addiction may be associated with weakened cognitive beliefs about the fairness and predictability of the world (BJW), which in turn may be associated with diminished subjective experience of life as meaningful and purposeful, which may ultimately be associated with impaired capacity for resilient adaptation to adversity. This sequential model positions BJW as a more distal cognitive factor and meaning in life as a more proximal experiential variable in the proposed pattern of associations from addiction to resilience. Therefore, we hypothesize:

*H4*: Belief in a just world and meaning in life sequentially mediate the relationship between short-form video addiction and psychological resilience (i.e., Video → BJW → Life → PR).

**Figure 1:**
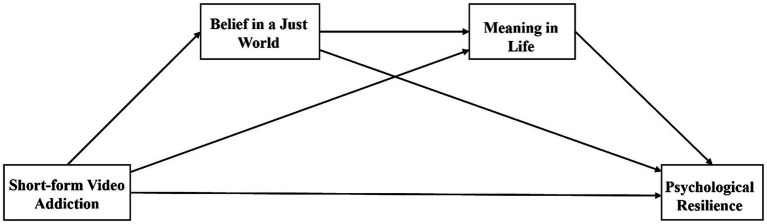
The proposed theoretical model.

Based on the aforementioned theoretical analysis and hypotheses, the present study proposes a serial mediation model to examine the integrated sequential pattern of associations through which belief in a just world and meaning in life may link short-form video addiction to psychological resilience among college students (see Figure 1).

### 1.3 The present study

The present study aims to systematically examine the pathways through which short-form video addiction is associated with psychological resilience among Chinese college students, using a serial mediation model in which BJW and meaning in life serve as sequential mediators. This investigation contributes to the existing literature in several ways. First, it applies just world theory to the domain of digital addiction, examining how addictive engagement with algorithmically curated content platforms may be associated with cognitive beliefs about the world’s fairness. Second, it integrates cognitive and existential theoretical perspectives within a single framework, offering a more comprehensive account of the pathways linking digital addiction to adaptive functioning than either perspective alone can provide. Third, the identification of multiple indirect pathways has practical significance for intervention design, suggesting that preventive and remedial efforts can target distinct psychological processes at different levels of the cognitive-experiential hierarchy. The large sample (*N* = 4,456) drawn from five universities representing different tiers of the Chinese higher education system ensures adequate statistical power for detecting mediation effects, and the focus on Chinese college students addresses a population that is both highly exposed to short-form video platforms and navigating a critical developmental period during which resilience is especially consequential.

## 2 Method

### 2.1 Participants and procedure

Participants were recruited from five universities in Sichuan Province, China, using a stratified random sampling strategy designed to ensure representation across different tiers of the Chinese higher education system. The participating institutions included one Project 985 university, two Double First-Class universities, and two regular undergraduate institutions, thereby capturing a range of academic environments and student populations. The target sample size was determined through an *a priori* power analysis using G*Power 3.1 (Faul et al., 2009). Based on a medium effect size (*f*^2^ = 0.15), *α* = 0.05, and desired power of 0.95 for a regression model with four predictors, the minimum required sample was 146. However, given the complexity of the serial mediation model, the anticipated need for bootstrap-based inference, and the possibility of missing data, a substantially larger target of 4,500 participants was established to ensure robust estimation of all direct and indirect effects.

Data were collected between November and December 2025 via a widely used online survey platform (Wenjuanxing). Recruitment notices were disseminated through university academic affairs systems and campus psychological health centers, clearly describing the study’s purpose, the voluntary and anonymous nature of participation, and the estimated completion time. Prior to completing the survey, all participants reviewed and electronically signed an informed consent form that explained data usage, confidentiality protections, and the right to withdraw at any time without penalty. Two attention-check items (e.g., “Please select ‘Strongly Agree’ for this item”) were embedded at different points in the survey to detect careless or inattentive responding. The average completion time was approximately 15–20 min.

An initial pool of 4,680 responses was collected. Following the application of predetermined exclusion criteria—completion time under 5 min (indicating rushed or random responding), failure on either attention-check item, and evidence of systematic patterned responding (e.g., selecting the same response option for all items within any given scale)—224 responses were removed, yielding 4,456 valid questionnaires (effective response rate = 95.2%). The final sample comprised 2,134 males (47.9%) and 2,322 females (52.1%), with ages ranging from 18 to 25 years (*M* = 20.34, *SD* = 1.52). The distribution across academic years was as follows: freshmen (*n* = 1,245, 27.9%), sophomores (*n* = 1,167, 26.2%), juniors (*n* = 1,089, 24.4%), and seniors (*n* = 955, 21.4%). Regarding academic disciplines, 45.4% (*n* = 2,023) were enrolled in STEM fields, 39.4% (*n* = 1,756) in humanities and social sciences, and 15.2% (*n* = 677) in arts and sports programs. In terms of short-form video usage, participants reported a mean daily viewing time of 2.8 h (*SD* = 1.6); the most frequently used platforms included Douyin (89.3%), Bilibili (56.7%), and Kuaishou (34.2%).

This study was reviewed and approved by the Ethics Review Committee of the Sichuan Provincial Psychological Association (Approval No. 2025032). All procedures were conducted in accordance with the ethical principles of the Declaration of Helsinki (World Medical Association, 2013) and the ethical guidelines for psychological research established by the Chinese Psychological Society.

### 2.2 Measures

All instruments had been previously validated in Chinese populations. Unless otherwise noted, scales used five-point Likert response scales (1 = strongly disagree to 5 = strongly agree). The Meaning in Life Questionnaire employed a seven-point Likert response scale (1 = absolutely untrue to 7 = absolutely true). Scale scores were computed as the sum of constituent items unless otherwise noted, with higher scores indicating greater levels of the respective construct.

#### 2.2.1 Short-form video addiction

Short-form video addiction was assessed using a 20-item scale adapted for the short-form video context. The scale was developed following the component model of behavioral addiction (Griffiths, 2005), which identifies six core features of addictive behavior: salience, tolerance, mood modification, conflict, withdrawal, and relapse. Items were adapted to specifically reference short-form video usage [e.g., “I need to spend increasingly more time watching short videos to feel satisfied” (tolerance); “I feel anxious and restless when I cannot watch short videos” (withdrawal); “Watching short videos has interfered with my academic work” (conflict); “Watching short videos has become the most important activity in my life” (salience); “I watch short videos to escape negative emotions” (mood modification); “I have repeatedly tried to reduce my short video use but failed” (relapse)]. This adaptation strategy is consistent with approaches used in recent Chinese short-form video addiction research, in which established addiction frameworks have been modified to capture the distinctive features of short-form video platforms (Mu et al., 2022; Ye et al., 2022). The scale demonstrated excellent internal consistency in the present sample (Cronbach’s *α* = 0.93), and confirmatory factor analysis indicated adequate model fit: *χ*^2^/df = 3.24, CFI = 0.96, TLI = 0.95, RMSEA = 0.048, SRMR = 0.042, with all standardized factor loadings exceeding 0.60.

#### 2.2.2 Belief in a just world

BJW was measured using a 12-item scale based on Dalbert’s (1999) General Belief in a Just World Scale and its validated Chinese adaptation (Su et al., 2012). The scale assesses the general cognitive belief that the world is fundamentally fair and that individuals typically receive the outcomes they deserve. Sample items include “I believe that the world is basically a just place” and “I think that people generally get what they deserve.” This scale has been widely used in Chinese samples and has demonstrated sound psychometric properties across multiple populations, including college students (Su et al., 2012; Wu et al., 2011). In the present sample, Cronbach’s α was 0.85. Confirmatory factor analysis yielded acceptable fit indices: *χ*^2^/df = 4.12, CFI = 0.98, TLI = 0.96, RMSEA = 0.052, SRMR = 0.031.

#### 2.2.3 Meaning in life

Meaning in life was assessed using the full 10-item Meaning in Life Questionnaire (MLQ; Steger et al., 2006), which evaluates two theoretically distinct dimensions of the meaning in life construct: presence of meaning (the degree to which individuals currently feel their lives are meaningful and purposeful; 5 items) and search for meaning (the degree to which individuals are actively seeking meaning or purpose in their lives; 5 items). Together, these two dimensions provide a comprehensive assessment of individuals’ overall meaning in life experience, capturing both its experiential and motivational aspects. As theorized by Steger et al. (2006) and subsequent empirical work, both dimensions are relevant to psychological adaptation: presence of meaning is consistently and positively associated with well-being and resilience, while search for meaning reflects an ongoing motivational orientation toward purposeful engagement (Park, 2010). Using the total MLQ score as the operationalization of meaning in life in the mediation model is consistent with treating meaning in life as a unitary psychological resource within the COR framework, while the two-factor structure of the instrument is preserved in the measurement model. The scale uses a seven-point Likert response format (1 = absolutely untrue to 7 = absolutely true). The Chinese version of the MLQ has been validated in multiple studies with college student samples and demonstrates strong factorial validity, with the original two-factor structure consistently replicated (Jiang et al., 2016; Liu and Gan, 2010; Wang and Dai, 2008). Sample items include “I understand my life’s meaning” (presence) and “I am searching for something that makes my life feel meaningful” (search). In the current sample, Cronbach’s *α* was 0.89, and confirmatory factor analysis of the two-factor structure indicated excellent fit: *χ*^2^/df = 2.87, CFI = 0.99, TLI = 0.98, RMSEA = 0.043, SRMR = 0.024, with all standardized factor loadings exceeding 0.60.

#### 2.2.4 Psychological resilience

Psychological resilience was assessed using the 25-item Connor-Davidson Resilience Scale (CD-RISC; Connor and Davidson, 2003). The CD-RISC is one of the most widely used resilience measures internationally and has been identified as possessing the strongest psychometric properties among existing resilience scales in a comprehensive methodological review (Windle et al., 2011). The Chinese version was developed and validated by Yu and Zhang (2007), who identified a three-factor structure (tenacity, strength, and optimism) with a total scale reliability coefficient of 0.91 in a Chinese community sample. Subsequent validation studies have confirmed the scale’s robust psychometric properties across diverse Chinese populations, including adolescents (Yu et al., 2011) and military personnel (Xie et al., 2016). Items assess the capacity to adapt to change, cope with adversity, maintain goal orientation, and recover from setbacks (e.g., “I am able to adapt when changes occur”; “Coping with stress strengthens me”; “I tend to bounce back after illness or hardship”). In the present sample, Cronbach’s α was 0.92, and confirmatory factor analysis yielded adequate fit: *χ*^2^/df = 3.45, CFI = 0.97, TLI = 0.96, RMSEA = 0.046, SRMR = 0.038.

#### 2.2.5 Measurement model summary

To provide a comprehensive evaluation of the measurement model, composite reliability (CR) and average variance extracted (AVE) were computed for all four constructs in addition to Cronbach’s alpha. As shown in Table 1, all CR values exceeded the recommended threshold of 0.70 and all AVE values exceeded the recommended threshold of 0.50 (Fornell and Larcker, 1981; Hair et al., 2010), supporting the convergent validity of each scale. Discriminant validity was formally assessed using the Fornell-Larcker criterion (Fornell and Larcker, 1981): the square root of the AVE (√AVE) for each construct, shown on the diagonal of Table 1, exceeded all inter-construct correlations in the corresponding row and column. This criterion was most stringently tested by the strong correlation between meaning in life and psychological resilience (*r* = 0.61); the √AVE values for both constructs nonetheless exceeded this coefficient, confirming that these two instruments assess empirically distinguishable psychological constructs despite their conceptual relatedness. Taken together, these results support the adequacy of the measurement model and indicate that common method variance did not artifactually merge conceptually distinct constructs.

**Table 1:** Measurement model: internal consistency, convergent validity, and discriminant validity.

Construct	Items	α	CR	AVE	1	2	3	4
1. Short-form video addiction	20	0.93	0.95	0.86	**0.93**			
2. Belief in a just world	12	0.85	0.89	0.63	−0.25	**0.79**		
3. Meaning in life	10	0.89	0.91	0.77	−0.21	0.27	**0.88**	
4. Psychological resilience	25	0.92	0.94	0.85	−0.21	0.28	0.61	**0.92**

*N* = 4,456. *α*, Cronbach’s alpha; CR, composite reliability; AVE, average variance extracted. Bold diagonal values represent the square root of AVE (√AVE) for each construct; off-diagonal values represent bivariate inter-construct correlations. The Fornell-Larcker criterion for discriminant validity is satisfied when the √AVE for each construct exceeds all correlations in its corresponding row and column. Convergent validity is supported when AVE ≥ 0.50 and CR ≥ 0.70. ***p < 0.001.

### 2.3 Data analysis

All analyses were conducted using SPSS 27.0 and the PROCESS macro version 4.2 (Hayes, 2022). The analytic procedure comprised two stages. In the preliminary stage, data were screened for missing values, multivariate outliers, and distributional assumptions. Missing values constituted less than 2% of all data points and were addressed using the expectation–maximization algorithm. Multivariate outliers were identified using Mahalanobis distance (critical value: *χ*^2^(4) = 18.47, *p* < 0.001); fewer than 1% of cases were flagged and removed. Distributional properties were evaluated using skewness and kurtosis statistics, with absolute values below 2 and 7, respectively, considered acceptable for parametric analysis (Curran et al., 1996). Common method bias was assessed using Harman’s single-factor test. Descriptive statistics, bivariate Pearson correlations, and multicollinearity diagnostics (variance inflation factors) were computed for all study variables.

In the primary analysis stage, the serial mediation model was tested using PROCESS Model 6 (Hayes, 2022). Short-form video addiction was specified as the independent variable (*X*), BJW as the first mediator (*M*₁), meaning in life as the second mediator (*M*₂), and psychological resilience as the dependent variable (*Y*). The statistical significance of all indirect effects was evaluated using bias-corrected bootstrap confidence intervals based on 5,000 resamples; an indirect effect was considered significant if its 95% confidence interval did not contain zero (Hayes, 2022). Four categories of effects were examined: (a) the total effect of *X* on *Y* (*c*), (b) the direct effect of *X* on *Y* controlling for both mediators (*c’*), (c) the total indirect effect, and (d) three specific indirect effects: Ind1 (Video → BJW → PR), Ind2 (Video → Life → PR), and Ind3 (Video → BJW → Life → PR). To evaluate the robustness of the hypothesized mediator sequence, an alternative model reversing the order of the two mediators (Video → Life → BJW → PR) was also estimated and compared with the hypothesized model on the basis of the serial mediation effect magnitude and theoretical coherence.

## 3 Results

### 3.1 Preliminary analyses

Descriptive statistics and bivariate correlations among the study variables are presented in Table 2. All variables exhibited acceptable distributional properties for parametric analysis. Skewness values ranged from −0.31 to −0.15 and kurtosis values ranged from 0.33 to 0.67, well within the recommended thresholds of |skewness| < 2 and |kurtosis| < 7 (Curran et al., 1996). Harman’s single-factor test revealed that the first unrotated factor accounted for 28.3% of total variance, substantially below the conventional 50% threshold, indicating that common method bias was not a serious concern in the present data (Podsakoff et al., 2003).

**Table 2:** Descriptive statistics and bivariate correlations among study variables (*N* = 4,456).

Variable	*M*	*SD*	Skewness	Kurtosis	1	2	3	4
1. Video	43.21	13.45	−0.23	0.67	—			
2. BJW	47.82	7.33	−0.15	0.42	−0.25***	—		
3. Life	48.76	7.91	−0.31	0.58	−0.21***	0.27***	—	
4. PR	51.34	10.59	−0.18	0.33	−0.21***	0.28***	0.61***	—

Video, short-form video addiction; BJW, belief in a just world; Life, meaning in life; PR, psychological resilience. ***p < 0.001.

The correlation matrix revealed patterns consistent with the hypothesized model. Short-form video addiction was negatively correlated with BJW (*r* = −0.25), meaning in life (*r* = −0.21), and psychological resilience (*r* = −0.21, all *p* < 0.001). BJW was positively correlated with both meaning in life (*r* = 0.27) and resilience (*r* = 0.28, both *p* < 0.001). The strongest correlation was between meaning in life and resilience (*r* = 0.61, *p* < 0.001). As noted in the Method section, the Fornell-Larcker criterion confirmed adequate discriminant validity between these constructs. All variance inflation factors were below 2.5, indicating no multicollinearity concerns.

### 3.2 Serial mediation analysis

**Figure 2:**
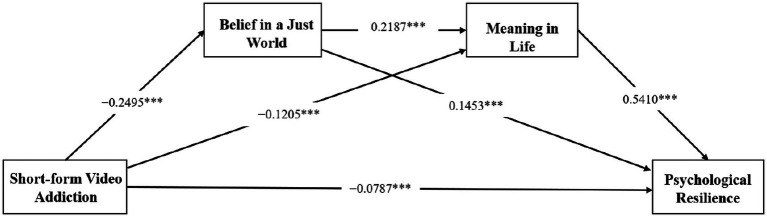
The final serial mediation model with standardized path coefficients. Standardized path coefficients (*β*) are presented. ***p < 0.001.

Results of the serial mediation model (PROCESS Model 6) are summarized in Tables 3, 4 and depicted in Figure 2.

**Table 3:** Regression path coefficients in the serial mediation model.

Predictor	*B*	*SE*	*t*	*β*	*R* ^2^	*F*
Outcome: BJW						
Video	−0.1292	0.0075	−17.19***	−0.2495	0.0622	295.61***
Outcome: life meaning						
Video	−0.0677	0.0084	−8.10***	−0.1205	0.0755	181.84***
BJW	0.2372	0.0161	14.70***	0.2187		
Outcome: psychological resilience						
Video	−0.0731	0.0114	−6.41***	−0.0787	0.3796	908.18***
BJW	0.2607	0.0224	11.64***	0.1453		
Life	0.8952	0.0203	44.06***	0.5410		

*N* = 4,456. Video, short-form video addiction; BJW, belief in a just world; Life, meaning in life. ***p < 0.001.

**Table 4:** Total, direct, and indirect effects of short-form video addiction on psychological resilience.

Effect	*B*	*SE*	*t*	*p*	95% CI
Total effect (*c*)					
Video → PR	−0.1948	0.0136	−14.31	< 0.001	[−0.2215, −0.1681]
Direct effect (*c’*)					
Video → PR	−0.0731	0.0114	−6.41	< 0.001	[−0.0955, −0.0507]
Indirect effects	*B*	Boot *SE*			Boot 95% CI
Total indirect	−0.1217	0.0096	—	—	[−0.1413, −0.1027]
Ind1: Video → BJW → PR	−0.0337	0.0043	—	—	[−0.0426, −0.0254]
Ind2: Video → Life → PR	−0.0606	0.0082	—	—	[−0.0769, −0.0443]
Ind3: Video → BJW → Life → PR	−0.0274	0.0031	—	—	[−0.0337, −0.0215]

Bootstrap sample size = 5,000. CI, bias-corrected confidence interval. Ind, specific indirect effect. Completely standardized total indirect effect = −0.1310, 95% CI [−0.1509, −0.1115].

As shown in Table 3, short-form video addiction significantly and negatively predicted BJW (*β* = −0.2495, *p* < 0.001), accounting for 6.22% of its variance. When both video addiction and BJW were entered as predictors of meaning in life, both contributed significantly (*β* = −0.1205 and *β* = 0.2187 respectively, both *p* < 0.001), jointly explaining 7.55% of variance. In the full model predicting resilience, all three predictors remained significant (all *p* < 0.001), collectively explaining 37.96% of variance in psychological resilience.

As shown in Table 4, the total effect of short-form video addiction on psychological resilience was significant and negative (completely standardized effect = −0.2096, *p* < 0.001), supporting H1. After controlling for both mediators, the direct effect remained significant but substantially attenuated (−0.0787, *p* < 0.001), indicating partial mediation. The direct effect accounted for 37.5% of the total effect.

The total indirect effect was significant [completely standardized = −0.1310, 95% CI (−0.1509, −0.1115)], accounting for 62.5% of the total effect. All three specific pathways were significant: through BJW alone (Ind1: 17.3% of total effect, supporting H2), through meaning in life alone (Ind2: 31.1% of total effect, supporting H3), and through the serial chain from BJW to meaning in life (Ind3: 14.1% of total effect, supporting H4). The second pathway (Ind2) was the largest specific indirect effect. All the standardized path coefficients are shown in Figure 2.

An alternative model reversing the mediator sequence (Video → Life → BJW → PR) was estimated to evaluate the robustness of the hypothesized ordering. Both models yielded identical total effect, direct effect, and total indirect effect, as well as identical *R*^2^ for resilience (0.3796). However, the hypothesized model (Video → BJW → Life → PR) produced a serial mediation effect [−0.0274, 95% CI (−0.0337, −0.0215)] approximately 5.5 times larger than the alternative model [−0.0050, 95% CI (−0.0067, −0.0035)]. This substantial difference, combined with theoretical support for the BJW → Life direction (Heintzelman and King, 2014), supports retention of the hypothesized model. All four hypotheses received empirical support.

## 4 Discussion

### 4.1 Summary of main findings

The present study investigated the psychological pathways underlying the association between short-form video addiction and psychological resilience among 4,456 Chinese college students, with specific attention to the serial mediating roles of belief in a just world and meaning in life. Four principal findings emerged. First, short-form video addiction was significantly and negatively associated with psychological resilience, with a completely standardized total effect of −0.21. Second, all three hypothesized indirect pathways were statistically significant: through BJW alone (17.3% of total effect), through meaning in life alone (31.1% of total effect), and through the sequential chain from BJW to meaning in life (14.1% of total effect). Collectively, these indirect pathways accounted for 62.5% of the total association between video addiction and resilience, leaving 37.5% attributable to the direct association and unmeasured factors. Third, meaning in life emerged as the strongest proximal correlate of psychological resilience, with a standardized coefficient (*β* = 0.541) approximately 3.7 times larger than that of BJW and nearly seven times larger than the direct association of video addiction with resilience. Fourth, comparison with an alternative model reversing the mediator sequence confirmed that the BJW → meaning in life ordering produced a serial mediation association approximately 5.5 times larger than the reverse, a pattern consistent with a hierarchical model in which cognitive worldviews are positioned as antecedents to existential experience in the proposed sequential pathway.

### 4.2 Theoretical implications

#### 4.2.1 Integrating cognitive and existential perspectives on digital addiction

A key theoretical contribution of the present study lies in the integration of cognitive and existential psychological perspectives within a single empirical framework for understanding how digital addiction is associated with adaptive functioning. The present findings suggest that BJW and meaning in life function not as independent psychological resources but as hierarchically ordered components of a cognitive-existential system that may be associated with disruption from addictive digital media use.

The serial mediation pathway (Video → BJW → Life → PR) provides evidence consistent with a sequential association pattern in which weakened cognitive beliefs about the world’s fairness are associated with diminished existential meaning, which in turn is associated with lower resilient adaptation. This finding extends previous work by Tian et al. (2022), who demonstrated that meaning in life mediated the relationship between BJW and mental toughness in adolescent athletes (a construct related to but conceptually distinct from psychological resilience, as discussed in Section 1.2.4), by identifying short-form video addiction as a specific behavioral antecedent that may be associated with this BJW → meaning in life association pattern. Additionally, the present results converge with recent findings by Zhou and Toikko (2025), who reported that the presence of meaning in life mediated the relationship between BJW and mental health outcomes among Chinese young adults. Our study extends this work by examining a specific upstream behavioral variable and by demonstrating its associations with resilience specifically.

The finding that the BJW → meaning in life direction produced a substantially stronger serial mediation association than the reverse (−0.0274 vs. −0.0050) is consistent with a hierarchical cognitive-existential model in which foundational worldviews provide epistemic scaffolding for constructing existential meaning. This directional relationship aligns with Heintzelman and King’s (2014) theoretical argument that perceptions of coherence and predictability—core psychological functions of BJW—may serve as preconditions for experiencing life as meaningful. It also aligns with the cognitive-experiential framework proposed by Beck (1976), in which core cognitive schemas are associated with emotional experience and behavioral adaptation.

#### 4.2.2 Short-form video addiction as a distinctive digital threat

The present findings contribute to a growing body of literature documenting associations between short-form video addiction and psychological outcomes among college students. While prior research has primarily examined its relationships with academic procrastination (Xie et al., 2023), sleep quality (Zhao and Kou, 2024), and learning motivation (Ye et al., 2022), the current study extends this work to psychological resilience—a broader adaptive capacity with implications for long-term mental health and developmental adjustment. The significant negative association between video addiction and resilience (standardized total effect = −0.21) is broadly consistent with effect sizes observed in related domains of digital addiction, and the large sample size provides confidence in the reliability of this estimate.

Of particular theoretical interest is the significant indirect pathway through BJW, which points to a theoretically proposed process involving the content environment of algorithmically curated short-form video platforms. The present study did not directly measure content exposure types or users’ subjective experience of algorithmic curation; however, emerging empirical evidence provides grounding for the proposed pathway. From a cultivation theory perspective—which has been empirically extended to social media contexts, with meta-analytic evidence indicating that specific content exposure produces larger belief-shaping effects than general social media use, and that effects are elevated among younger and Asian samples (Hermann et al., 2023)—sustained consumption of specific content categories on short-form video platforms may be associated with corresponding shifts in worldview beliefs such as BJW. Consistent with this view, passive social media behaviors have been empirically associated with heightened awareness of undesirable events in others’ lives, which in turn has been linked to lower perceptions of fairness for others (Shin and Hampton, 2023). Research has further demonstrated that exposure to wealth-displaying content on social media triggers upward social comparison and heightened perceptions of relative unfairness (Yan et al., 2025; Yang et al., 2025)—processes conceptually related to BJW erosion, insofar as both involve perceptions that one’s situation is unjustly inferior to that of comparable others. Importantly, the relationship between social media use and BJW appears sensitive to the nature of engagement: general adaptive social media engagement has been found to be positively associated with personal BJW (Zhou et al., 2024), while the passive, compulsive consumption pattern characteristic of short-form video addiction may create the specific content exposure conditions most closely linked to BJW erosion. Together, these findings provide indirect empirical grounding for the proposed pathway, while underscoring that the direct test of content exposure as the operative mechanism—through designs that measure exposure type, frequency, and affective responses to specific content categories—remains a priority for future research.

The present findings further raise the question of whether the BJW-mediated pathway may represent a theoretically distinctive feature of algorithmically curated platforms relative to other digital media forms. As recent systematic reviews have documented, the algorithmic features of short-form video platforms—including highly personalized recommendation systems and engagement-optimized content delivery—may create conditions particularly conducive to compulsive use and sustained content immersion (Mu et al., 2022; Tian et al., 2023; Zhan and Zhu, 2025), which may simultaneously be associated with exposure to content patterns that shape users’ cognitive worldviews. However, the present study did not include a cross-media comparison condition, and the question of whether short-form video addiction produces BJW-related effects that are genuinely distinct from those associated with other forms of digital media use cannot be answered from the present data. Future research employing comparative designs across different platform types would be needed to evaluate this theoretical proposition.

#### 4.2.3 Meaning in life as a proximal mechanism for resilience

A particularly notable finding is the magnitude of meaning in life as a correlate of psychological resilience (standardized *β* = 0.5410), substantially exceeding the contributions of both BJW and the direct association of video addiction with resilience. This finding converges with Frankl’s (1985) foundational assertion that meaning represents a central determinant of psychological endurance and with recent empirical reviews documenting robust positive associations between meaning in life and resilience across diverse populations (Miao and Cao, 2024). A recent meta-analysis including 228 studies and 188,365 participants confirmed that meaning in life shows medium positive correlations with positive mental health indicators among college students (Zhan and Zhu, 2025), and an emerging body of work has begun to elaborate the self-regulatory pathways through which meaning is associated with resilience, including stress buffering, adaptive coping facilitation, and healthy behavior engagement (Miao and Cao, 2024). The present findings add to this literature by suggesting that meaning in life may function as a particularly strong proximal correlate linking short-form video addiction and resilience outcomes in a Chinese college student sample.

The centrality of meaning in life in the current model carries implications for the conceptualization of resilience itself. If meaning in life is a substantially stronger correlate of resilience than either cognitive worldviews or the direct association of addictive behavior, then resilience may be best understood not primarily as a set of coping skills or personality traits, but as a capacity that is fundamentally grounded in the experience of existential purpose. This interpretation is consistent with Park’s (2010) meaning-making model of coping, which posits that meaning represents a central process through which individuals achieve psychological adjustment following adversity, and with Masten’s (2001) characterization of resilience as arising from ordinary adaptive systems rather than extraordinary psychological capacities.

### 4.3 Practical implications

The identification of three distinct indirect association pathways is consistent with a multi-layered intervention framework addressing the co-occurrence of addictive short-form video use and reduced resilience at different levels of psychological functioning. It is important to acknowledge at the outset that, because the present data are cross-sectional and all associations are correlational, the proposed intervention strategies represent theoretically informed hypotheses rather than empirically validated clinical recommendations. The causal direction of the identified associations has not been established, and experimental or quasi-experimental research will be required to evaluate whether targeting these pathways produces improvements in resilience, reductions in addictive use, or both.

At the behavioral level, foundational efforts should focus on reducing problematic short-form video usage itself, as behavioral change represents a prerequisite for addressing the co-occurring psychological patterns identified in the present model. Targeted digital literacy education that explains how algorithmic recommendation systems function and how engagement-optimized content delivery may shape users’ perceptions of social reality could support students in developing more critical appraisals of platform-generated content, directly addressing the content exposure process theorized to underlie the BJW pathway. Such literacy-based approaches could be combined with time management strategies and structured encouragement of meaningful alternative activities—particularly those involving active, goal-directed engagement that may displace compulsive passive viewing while simultaneously supporting a sense of purpose. University counseling centers could integrate brief screening for short-form video addiction into routine mental health assessments, enabling early identification of students whose viewing patterns may place them at risk. Given that over 80% of Chinese college students regularly engage with short-form video platforms (Liu Z., 2019), preventive approaches should target the broader student population rather than only those manifesting clinical-level addiction.

At the cognitive level, the significant BJW mediation pathway suggests potential value in interventions designed to support fair-world appraisals that may co-occur with addictive viewing patterns. Media literacy programs that explicitly address how algorithmic curation may create content environments that overrepresent injustice, inequality, and aspirational wealth displays—contributing to worldview appraisals that do not accurately reflect the distribution of social reality—may be valuable for supporting more balanced BJW appraisals among heavy users. Cognitive restructuring techniques from cognitive-behavioral therapy could supplement media literacy by helping students develop more balanced evaluations of fairness in their broader social environment. Given the correlational nature of the present findings, the hypothesis that cognitive interventions targeting BJW would produce downstream improvements in meaning in life—as implied by the serial mediation pattern—requires experimental verification before cascading benefits can be claimed. The serial mediation pattern nonetheless raises the theoretical possibility that cognitive interventions targeting BJW could be associated with potential downstream improvements in meaning in life, representing one plausible mechanism for cascading benefits across these psychological processes—a hypothesis warranting investigation in future intervention trials.

At the existential level, the finding that meaning in life is the strongest correlate of resilience in the present model suggests that meaning-focused interventions warrant consideration in university mental health programming. Because the co-occurrence of lower meaning and addictive short-form video use is bidirectional in the present correlational data, meaning-focused interventions are most plausibly effective when integrated with—rather than delivered independently of—behavioral interventions directly targeting addictive use patterns. Approaches grounded in logotherapy principles (Frankl, 1985)—including values clarification exercises, purpose identification workshops, and structured opportunities for meaningful engagement—may support the development of a sense of existential purpose that is less dependent on passive digital consumption; however, their specific effectiveness for individuals with short-form video addiction has not been empirically established and warrants dedicated investigation. Evidence that mindfulness-based interventions can enhance meaning in life, and that presence of meaning mediates relationships between mindfulness and resilience (Hooker et al., 2018), suggests that mindfulness programs may also be relevant. An integrated approach addressing behavioral patterns, cognitive processes, and existential meaning concurrently is likely to be most effective, reflecting the multi-layered and potentially mutually reinforcing nature of the associations revealed by the present model; the efficacy of such an integrated program specifically for short-form video addiction, however, requires evaluation through controlled intervention research before practical recommendations can be advanced with confidence.

### 4.4 Limitations and future directions

Several limitations warrant consideration. Most fundamentally, the cross-sectional design limits causal inferences about the directionality of observed relationships. Although the theoretical framework and serial mediation results are consistent with the proposed sequence (Video → BJW → Life → PR), alternative orderings cannot be excluded. It is plausible that individuals with low resilience turn to short-form videos as an escapist coping mechanism, that diminished meaning in life precedes and drives addictive viewing, or that unmeasured third variables such as depression, neuroticism, or socioeconomic adversity simultaneously influence all variables in the model. These alternative interpretations are inherent to cross-sectional mediation designs and should be borne in mind when interpreting all reported associations. The modest *R*^2^ values for the mediator variables (6.22% for BJW, 7.55% for meaning in life) confirm that short-form video addiction accounts for only a small proportion of variance in these multiply determined constructs. This finding is theoretically expected rather than anomalous: the present study does not propose that short-form video addiction is the primary determinant of BJW or meaning in life, but rather that it represents one contributing factor within a broader nomological network. Numerous other variables—including personality characteristics (e.g., neuroticism, conscientiousness), early life experiences, social support, and general cognitive dispositions—likely account for substantially more variance in both constructs. The practical significance of the identified indirect pathways lies in the theoretically consistent patterning of associations and in the proportion of the total addiction-resilience association that is collectively accounted for (62.5%), rather than in the absolute R2 values for individual mediators. Nonetheless, the limited independent explanatory power of video addiction for BJW and meaning in life suggests that interventions focused solely on reducing video addiction may produce only modest changes in these constructs, and that addressing their broader determinants may be equally or more important for resilience promotion. Future research should employ longitudinal designs with multiple assessment waves to establish temporal precedence, cross-lagged panel analyses to disentangle reciprocal effects, and experimental or quasi-experimental interventions to strengthen causal inferences.

Second, all measures relied on self-report, introducing potential social desirability bias and shared method variance. While Harman’s single-factor test indicated that the first unrotated factor accounted for 28.3% of total variance—well below the 50% threshold—it is important to acknowledge that the Harman single-factor test is recognized as a conservative and relatively insensitive indicator of common method bias (Podsakoff et al., 2003), and its favorable result cannot fully exclude the possibility that shared method variance has inflated observed inter-construct correlations. This concern is most acute for the strong correlation between meaning in life and psychological resilience (*r* = 0.61): although the Fornell-Larcker criterion confirmed that these constructs are empirically distinguishable, shared method variance may nonetheless have contributed to the magnitude of this association. Social desirability bias may also have differentially affected responses across measures, with participants potentially underreporting addictive viewing patterns. Future studies would benefit substantially from incorporating objective usage data (e.g., device-recorded screen time logs), behavioral or informant-rated assessments of resilience, and multi-source measurement designs to complement self-report data and provide more conservative estimates of the identified associations.

Third, despite explaining 37.96% of resilience variance, the model leaves substantial variance unaccounted for. Important variables not included—such as social support, personality traits (particularly neuroticism and conscientiousness), self-efficacy, and emotion regulation capacity—likely contribute additional explanatory power and may moderate the identified pathways.

Fourth, the sample was limited to college students in Sichuan Province, which constrains generalizability at three distinct levels. Geographically, all participants were recruited from universities in a single inland province, and digital media use patterns, psychological characteristics, and educational cultures may differ between Sichuan and more economically developed coastal regions of China. At the population level, the findings apply specifically to college students during emerging adulthood and may not extend to adolescents—who represent another major short-form video user demographic at an earlier developmental stage—or to working adults and older populations, for whom the developmental salience of the examined constructs may differ considerably. At the cultural level, BJW is known to function differently across individualist and collectivist cultural contexts (Dalbert, 2001; Hafer and Sutton, 2016), and the sources and significance of meaning in life may vary by culture and developmental stage. The present findings should therefore be understood as specific to Chinese college students in a collectivist cultural context. Replications across diverse cultural populations—including individualist cultural settings and other East Asian societies—are needed to evaluate the cross-cultural robustness of the proposed mediation structure.

Fifth, the short-form video addiction scale used in the present study was adapted from Griffiths (2005) components model framework for the short-form video context, and exploratory factor analysis (EFA) was not conducted prior to confirmatory factor analysis (CFA). While the strong CFA fit indices (CFI = 0.96, TLI = 0.95, RMSEA = 0.048, SRMR = 0.042) and the firm theoretical grounding of the six-factor structure in established behavioral addiction theory provide empirical and conceptual support for the scale’s structural adequacy, the absence of a prior EFA represents a methodological limitation. Future research adapting addiction scales to new behavioral or cultural contexts is encouraged to employ split-sample EFA-CFA procedures to empirically verify the factor structure before proceeding to confirmatory testing.

## 5 Conclusion

The present study demonstrates that the relationship between short-form video addiction and psychological resilience among Chinese college students is substantially mediated by two interconnected psychological processes—weakened belief in a just world and diminished meaning in life—which together account for 62.5% of the total association. Beyond quantifying these pathways, the findings suggest a broader pattern: that problematic digital media consumption may be associated not only with behavioral disruption but also with changes in foundational cognitive assumptions and existential orientations that sustain adaptive capacity. The serial mediation chain from just world beliefs to meaning in life reveals that these associations occur in a hierarchical fashion, with distal cognitive beliefs relating to more proximal existential experiences. This cognitive-existential cascade framework offers a theoretically grounded basis for designing interventions that address not only addictive behavior but also the deeper psychological processes with which it is associated. As algorithmically curated digital environments become an increasingly dominant feature of young adults’ daily experience, understanding and protecting the psychological resources that underlie resilience—particularly the capacity to find life meaningful—represents an important priority for both research and practice.

## Data Availability

The raw data supporting the conclusions of this article will be made available by the authors, without undue reservation.
